# Biochemical and structural characterization of DNA ligases from bacteria and archaea

**DOI:** 10.1042/BSR20160003

**Published:** 2016-10-06

**Authors:** Giulia Pergolizzi, Gerd K. Wagner, Richard P. Bowater

**Affiliations:** * Department of Biological Chemistry, John Innes Centre, Norwich Research Park, Norwich NR4 7UH, U.K.; † Department of Chemistry, Faculty of Natural & Mathematical Sciences, King's College London, Britannia House, 7 Trinity Street, London SE1 1DB, U.K.; ‡ School of Biological Sciences, University of East Anglia, Norwich Research Park, Norwich NR4 7TJ, U.K.

**Keywords:** adenylate, antibacterial compounds, ATP, DNA ligase, enzyme inhibitors, β-NAD^+^

## Abstract

DNA ligases are enzymes that seal breaks in the backbones of DNA, leading to them being essential for the survival of all organisms. DNA ligases have been studied from many different types of cells and organisms and shown to have diverse sizes and sequences, with well conserved specific sequences that are required for enzymatic activity. A significant number of DNA ligases have been isolated or prepared in recombinant forms and, here, we review their biochemical and structural characterization. All DNA ligases contain an essential lysine that transfers an adenylate group from a co-factor to the 5′-phosphate of the DNA end that will ultimately be joined to the 3′-hydroxyl of the neighbouring DNA strand. The essential DNA ligases in bacteria use β-nicotinamide adenine dinucleotide (β-NAD^+^) as their co-factor whereas those that are essential in other cells use adenosine-5′-triphosphate (ATP) as their co-factor. This observation suggests that the essential bacterial enzyme could be targeted by novel antibiotics and the complex molecular structure of β-NAD^+^ affords multiple opportunities for chemical modification. Several recent studies have synthesized novel derivatives and their biological activity against a range of DNA ligases has been evaluated as inhibitors for drug discovery and/or non-natural substrates for biochemical applications. Here, we review the recent advances that herald new opportunities to alter the biochemical activities of these important enzymes. The recent development of modified derivatives of nucleotides highlights that the continued combination of structural, biochemical and biophysical techniques will be useful in targeting these essential cellular enzymes.

## Abstract (precis)

DNA ligases seal breaks in DNA using a co-factor that supplies adenylate for the reaction. We review biochemical and structural characterization of DNA ligases from bacteria and archaea, highlighting novel co-factor variants that act as inhibitors and/or non-natural substrates of the enzymes.

## DNA LIGASES

The family of enzymes classified as nucleotidyl transferases play important roles in the metabolism of nucleic acids due to their ability to transfer phosphorus-containing groups between biomolecules. An important member of this family are the DNA ligases, which are essential to all living cells due to their roles in DNA replication, repair and recombination [1–7]. DNA ligases participate in a range of cellular reactions, with their role particularly well characterized for the joining of Okazaki fragments that are synthesized on the lagging (discontinuous) strand during replication. The biochemical activity of DNA ligases results in the sealing of breaks between 5′-phosphate and 3′-hydroxyl termini within a strand of DNA. DNA ligases have been differentiated as being adenosine-5′-triphosphate (ATP)-dependent (EC 6.5.1.1) or NAD^+^-dependent (EC 6.5.1.2) depending on the co-factor (or co-substrate) that is used during their reaction [2,3,8]. Typically, more than one type of DNA ligase is found in each organism [1–3,6,9,10]. Here, we review current biochemical and structural knowledge of these enzymes from bacteria and archaea. In addition, we go on to highlight modifications of co-factors that alter the biochemical activities of these important enzymes, with a particular focus on altered forms of β-nicotinamide adenine dinucleotide (β-NAD^+^). Recent developments in identifying such compounds as selective inhibitors of a range of NAD^+^-dependent enzymes is encouraging for their development as potential drug candidates [8,11,12].

DNA ligases from several sources were first isolated by several research groups during the 1960s and their reaction mechanisms were soon elucidated [13]. It was quickly realized that DNA ligases are extremely useful tools in molecular biological manipulations of DNA, which has led to their widespread usage in biotechnological applications [7,14,15]. In the case of both ATP-dependent DNA ligases (ADLs) and NAD^+^-dependent DNA ligases (NDLs), the joining of DNA breaks occurs via a ping-pong mechanism and the biochemical details are well-characterized for many DNA ligases [1–7]. (For NDLs, the reaction mechanism is discussed further below in reference to Figure 5.) Briefly, in the first interaction between the enzyme and co-factor, β-NAD^+^ (or ATP) binds to the active site of the enzyme; there, a nucleophilic lysine residue attacks the phosphate of the adenosine in the co-factor with subsequent release of the leaving group, β-nicotinamide mononucleotide (β-NMN) for NDLs (or PP_i_ for ADLs). After the positioning of the DNA nick in the proximity of the adenylated-ligase intermediate, the nucleophilic phosphate in the 5′ position of the DNA attacks the phosphoramidate bond to form an adenylated-DNA intermediate. Finally, the nucleophilic hydroxyl group in the 3′ position of the DNA attacks the new pyrophosphate bond with formation of a phosphodiester bond between the 5′ and 3′ positions of the DNA and release of AMP. As will be discussed within this review, structural studies using a range of enzymes have demonstrated that large-scale conformational movements take place for ligation reactions performed by NDLs and ADLs.

**Figure 1:**
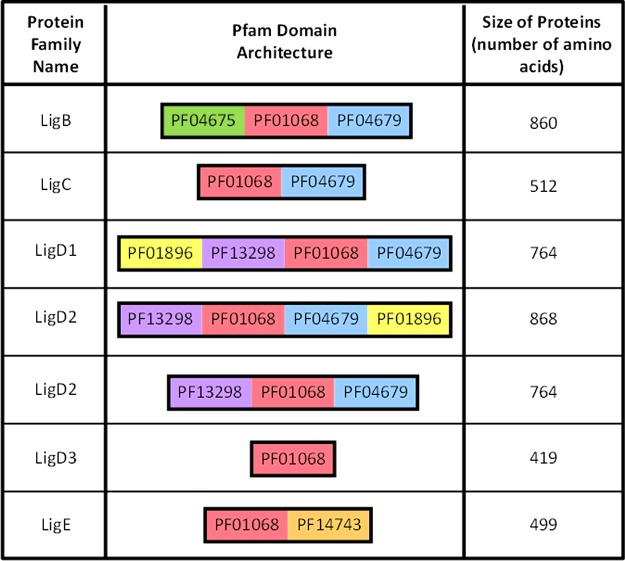
Conserved domains within the ATP-dependent DNA ligases from bacteria and archaea Schematic diagram indicating the position of conserved domains within the ATP-dependent DNA ligases identified in bacterial and archaeal genomes. Only protein domains matching to Pfam families [16] are referred to and polypeptide regions with no match are not depicted. Domains are not shown to scale. Sizes refer to numbers of amino acids in the largest protein within each domain architecture according to the Pfam database. Note that two related forms of LigD2 are observed within sequence databases. Details are adapted from [10]: Williamson, A., Hjerde, E. and Kahlke, T. (2016) Analysis of the distribution and evolution of the ATP-dependent DNA ligases of bacteria delineates a distinct phylogenetic group ‘Lig E'. Mol. Microbiol. **99**, 274–290.

**Figure 2:**
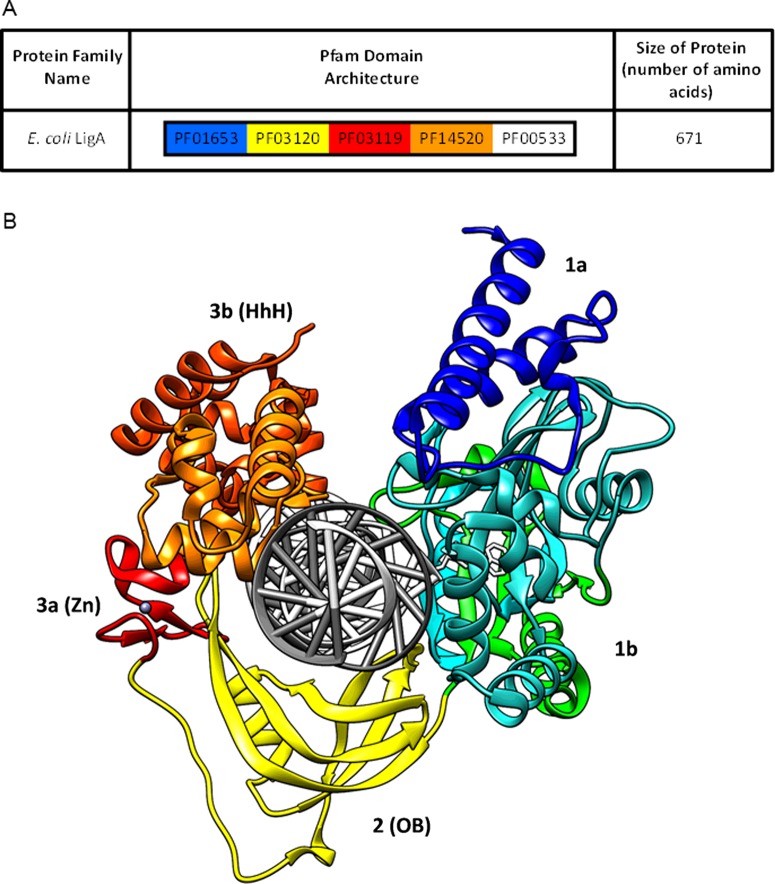
Conserved domains within the NAD^+^-dependent DNA ligases from bacteria (**A**) Schematic diagram indicating the approximate size and position of domains conserved within the NAD^+^-dependent DNA ligase (LigA) identified in *E. coli*. Only protein domains matching to Pfam families [16] are included and polypeptide regions with no match are not depicted. Domains are not shown to scale. Note that these Pfam domains are present in the majority of bacterial NAD^+^-dependent DNA ligases, though some contain additional Pfam domains that are not present in *E. coli* LigA. (**B**) High-resolution structure of *E. coli* LigA in complex with nicked adenylated DNA from PDB 2OWO [55], visualized by UCSF Chimera. The various domains are indicated by different colours and relate to Pfam domains as follows: subdomain 1a (light blue) and subdomain 1b (green/cyan) is equivalent to PF01653; OB-fold/domain 2 (yellow) is equivalent to PF03120; subdomain 3a (red) is equivalent to PF03119; subdomain 3b (orange) is equivalent to PF14520; the adenylated-DNA molecule is shown in grey. Note that the BRCA1 C-terminus (BRCT) domain (equivalent to PF00533) is not resolved in the crystallographic structure.

Although they all perform the same biochemical reaction, DNA ligases have been identified with a range of structures that incorporate a core ‘adenylation domain’ linked to other well-defined domains (Figures 1 and 2) [3,5–7]. The adenylation domains of ADLs and NDLs perform a similar enzymatic reaction, but they have key differences within their structures, as indicated by their presence as distinct domains within the Pfam database of proteins [16]. To highlight the key similarities and differences across ADLs and NDLs, we will briefly review the structural knowledge that informs biochemical function for the two classes of DNA ligases from bacteria and archaea. Notably, the evolutionary relationship between archaea and eukaryotes has recently been re-evaluated [17–19]. It is now clear that many genes have been transferred ‘horizontally’ from and into archaeal genomes, and this is almost certainly true for genes that encode DNA ligases. Thus, an evolutionary understanding of these genes may not be straightforward and it may be more useful for certain types of organisms than others. Nonetheless, detailed phylogenetic studies of essential cellular genes such as DNA ligases may offer useful information in relation to the evolution of eukaryotic cells, but this potential will not be considered in detail within this review.

## STRUCTURE AND FUNCTIONS OF ATP-DEPENDENT DNA LIGASES

ADLs occur in the genomes of all domains of life [1–3,6,9,10]. Viruses contain a diverse range of ADLs, whereas eukaryotes contain three main types of ADLs, although mitochondrial and nuclear localization signals direct some versions of the proteins to specific organelles [20]. Biochemical and structural details of DNA ligases from different organisms have been reviewed [1,3–7,9,20]. In this review we focus attention on DNA ligases from bacteria and, to a limited extent, archaea.

ADLs encoded within the genomes of archaea are relatively homologous in size and sequence, whereas those from bacteria have a wider diversity of total size and domain arrangements [2,10,14]. In the majority of archaea studied to date, the only functional genes for DNA ligases encode proteins that are of the ATP-dependent type, so the proteins they encode participate in all aspects of DNA metabolism. By contrast, where ADLs are found in bacteria, it is always in addition to, rather than instead of, NDLs and it is the NAD^+^-dependent versions that are essential [21], which we discuss below in more detail. The physiological requirements for so many different bacterial ADLs are not fully clear, though they appear to be not essential and are sometimes only expressed under particular growth conditions [2,22–26]. Furthermore, significant numbers of bacteria do not possess any genes for ADLs [2]. High-resolution structures exist for several ADLs from archaea [27–33] and bacteria [34,35].

In addition to their core adenylation domain, many ADLs include extra domains and motifs that enhance DNA binding, bring additional enzymatic activities or link them to macromolecular complexes that are important in specific aspects of DNA metabolism. With this knowledge it is not surprising that ADLs undergo large-scale conformational movements during the DNA ligation reactions. To assist comparison of the different types of ADLs (Figure 1), we follow the approach of Williamson et al*.* [10] and use the terminology from the Pfam database of proteins [16]. Distinct domains have a specific Pfam identifier, and those identified in ADLs are often found in other proteins involved in DNA metabolism. Note that PF01896 corresponds to DNA primase, which synthesizes short fragments of nucleic acid, usually RNA; PF01896 is not always recognized within DNA ligase sequences in the Pfam database, but these regions are usually annotated region as the Pol domain of LigD, which corresponds to this domain [10]. From this type of analysis of protein families it becomes clear that ADLs have significant diversity in their domains and in the way these are organized within a single polypeptide (Figure 1).

Where bacteria do encode ADLs within their genomes, the number of genes varies widely–though it is relatively consistent within each bacterial genus [2,10]. Three classes of these enzymes (LigB, LigC and LigD) appear to have descended from a common ancestor within bacteria. It is important to note that the various domains (and their corresponding biochemical activities) are arranged in different organizations within the LigD class of enzymes (Figure 1), which have been given different names [10]. This same recent comparison of the domain structure of different types of ADLs and their distribution among bacteria also delineated an additional group, termed LigE (Figure 1). These enzymes possess several unique features, suggesting that the genes encoding LigE-type proteins were horizontally transferred into bacteria in a separate event from other bacterial ADLs.

Functional characterization of the ADLs has delineated that the four classes of genes contain different organization of the structural domains found within the Pfam database [10]. The arrangement of the domains varies between species, but there are clear groupings of functional domains, as illustrated in Figure 1. The different domains are likely to promote interactions between the DNA ligase and other proteins, ensuring that the DNA-sealing polypeptide is targeted to specific processes or types of DNA ends. A number of observations suggest that ADLs assist the efficiency of DNA repair, with the best evidence being available for the non-homologous end-joining (NHEJ) system that repairs double-stranded breaks in DNA. The LigD proteins are required for efficient NHEJ that involve interactions with Ku proteins that also participate in NHEJ, but the LigC proteins also act as a back-up system [22–26,34,36–40]. Furthermore, the multiple enzymatic modules that are present within the large LigD proteins include a DNA-primase/polymerase domain and in some cases a phosphoesterase domain, and these activities are able to alter the structure and sequence of the ends before repairing them [41–45].

Bacterial ADLs have been demonstrated to have DNA end-joining activities by a variety of methods and assays, but the non-essential nature of these genes suggests that they are not functional as replicative DNA ligases. This is clearly different from the situation in the majority of archaea, where ADLs must participate in replication in each organism. The widespread presence of genes for ADLs in bacteria suggests that their protein products have important activities in DNA metabolism with their host cells. It seems likely that the various classes of ADLs have different physiological functions and, indeed, the function of a specific class may vary in different organisms. A number of possible functions have been suggested for these enzymes [10,22–26], but further research is required to clarify the usefulness of the various types of ADLs in different organisms.

## STRUCTURE AND FUNCTIONS OF NAD^+^-DEPENDENT DNA LIGASES

In contrast with the widespread presence of functional ADLs across cell types, the majority of functional NDLs have only been identified within the genomes of all bacteria and some viruses. In bacteria the genes are essential, presumably due to the requirement of their protein product for efficient DNA replication [21]. As discussed in detail below, the essential nature of the NDLs for bacterial viability make them a possible target for novel anti-bacterial drugs. Novel chemicals that are developed for such approaches also provide useful compounds for biochemical characterization of DNA ligases.

Some archaeal organisms encode NDLs, including *Methanomethylophilus alvus* [46] and, potentially, the recently discovered lokiarchaeota [19]. In contrast with the majority of archaea studied to date, genetic- and microbiology-based studies have confirmed the cellular requirement of an NDL homologue in the halophile *Haloferax volcanii* [47,48]. Phylogenetic analysis suggests the genes encoding these enzymes were most probably acquired as a result of horizontal gene transfer from bacteria [47,49]. Biochemical and structural characterization of NDLs encoded by archaeal genomes will illuminate the evolutionary history of these enzymes and help with the development of drugs that are specifically targeted against bacterial NDLs.

NDLs from different bacteria have been structurally characterized to better understand their activities and reaction mechanism, and to aid the development of possible inhibitors against these enzymes [3–5,7,8]. High levels of homology have been found between sequences of bacterial NDLs, showing highly conserved motifs and the same modular architecture. The sequence similarity with ADLs is quite poor, as expected because of the different co-factor; however, NDLs and ADLs share a similar biochemical reaction mechanism, as highlighted above [2,5,7,8].

The first crystallographic structure of an NDL was a partial structure that included the adenylation domain from the enzyme from *Bacillus stearothermophilus* [50]. Shortly after, a more complete and biochemically informative structure was obtained for the NDL from *Thermus filiformis* [51]. Other high-resolution structures have been resolved for the homologous enzymes from *Enterococcus faecalis* [52,53], *Mycobacterium tuberculosis* [54], *Escherichia coli* [55,56], *Haemophilus influenzae* [57], *Streptococcus pneumoniae* [56] and *Staphylococcus aureus* [58]. Many of the structures contain only fragments of the enzyme, but some have ligands incorporated. Comparison of the different structures reveals that the NDLs have a modular architecture consisting of four main domains (Figures 2A and 2B), which incorporate a number of domains from the Pfam database [16]. This rather complex conformation leads to several important features for the reaction mechanism of NDLs. Domain 1 (incorporating PF01653) is formed by two subdomains; subdomain 1a, at the N-terminus, binds the β-NMN moiety of β-NAD^+^, whereas subdomain 1b is the adenylation domain, or the nucleotidyltransferase domain (NTase), since it binds the AMP moiety of β-NAD^+^. Domain 2 is the oligonucleotide binding site (OB), analogous to PF03120. Domain 3 consists of subdomain 3a, a Cys_4_-type zinc finger (equivalent to PF03119), and subdomain 3b, containing a helix–hairpin–helix motif (HhH) (equivalent to PF14520). Domain 4, at the C-terminus, is a BRCT domain (breast cancer carboxy-terminal), analogous to PF00533. Note that, typically, the BRCT is not resolved in crystallographic structures of NDLs (e.g. as shown in Figure 2B).

The catalytic core of NDLs consists of subdomain 1b and domain 2 (OB-fold), flanked by N- and C-terminal domains arranged in a C-shape that clamps the double-stranded DNA [2–5,7,8]. Subdomain 1a has been shown to interact with the β-NMN moiety of β-NAD^+^ and, indeed, it is not present in ADLs [59]. Its presence is fundamental to initiate the ligation reaction of NDLs, but is not essential in the case of the interaction of preadenylated-enzyme with DNA; this means that subdomain 1a is required for the β-NAD^+^ binding and the adenylation step. From the crystallographic studies, it was possible to identify an ‘open’ enzyme conformation, in which β-NAD^+^ was bound to the solvent exposed NTase domain. Therefore, the closure of subdomain 1a to bury β-NAD^+^ puts β-NMN in an apical position as a leaving group during the nucleophilic attack of the lysine residue on the adenosine phosphate; the subdomain 1a has to swivel by almost 180° to switch from the ‘open’ to the ‘closed’ conformation [52].

Subdomain 1b contains the adenylation domain of NDLs, or the core of the DNA ligase. Several residues in the NTase have been found to be fundamental for the enzyme activity, with modifications bringing a considerable rate decrease or inactivation of the enzyme. The essential amino acids in the NTase, their interactions and their biochemical roles are summarized in Table 1 for the NDLs from *E. coli* [59,60] and *M. tuberculosis* [54,61]. The high conservation of NDLs is highlighted by the presence of similar residues in NDLs from the two organisms, with the only difference being that the stacking interaction to adenine occurs with Tyr-225 in EcligA, while it is with His-236 in MtLigA.

**Table 1:** Essential amino acids and their roles in the NTase domains of β-NAD^+^-dependent DNA ligases from *E. coli* [59,60] and *M. tuberculosis* [54,61] ‘Roles’ refer to the biochemical mechanism of DNA ligases, as shown in Figure 5.

	Residue	Interaction	Role
*E. coli*	K115	Part of KXDG conserved motif; nucleophilic attack to adenosine phosphate of β-NAD^+^	Formation of adenylated-ligase intermediate
*M. tuberculosis*	K123		
*E. coli*	D117	Part of KXDG conserved motif	Stabilization of active site
*M. tuberculosis*	D125		
*E. coli*	G118	Part of KXDG conserved motif	Stabilization of active site
*M. tuberculosis*	G126		
*E. coli*	E173	Coordination of O-2′ of the adenosine ribose in β-NAD^+^ and AMP	Involved in formation of adenylated-ligase intermediate and sealing of nick
*M. tuberculosis*	E184		
*E. coli*	K290	H-bonding with N-1 on adenine ring	Stabilization of adenine conformation
*M. tuberculosis*	K300		
*E. coli*	Y225	Stacking interaction with adenine ring	Stabilization of adenine conformation
*M. tuberculosis*	H236		
*E. coli*	E113	H-bonding with N-6 on adenine ring	Stabilization of adenine conformation
*M. tuberculosis*	E121		

Domain 2 contacts the double-stranded DNA, covers the nick and helps in positioning it into the NTase domain. Its action is supported by subdomain 3b, also referred to as HhH, which binds both DNA strands in a non-specific sequence manner across the minor groove. The OB-fold, NTase and HhH have been shown to clamp the double-strand DNA across 19 bp [55].

Subdomain 3a, the Cys_4_-type zinc finger, holds the OB-fold and HhH at the appropriate structural positions during DNA binding [51,55,62]. Finally, the C-terminal domain 4, BRCT, has a role for the DNA ligases that is still not fully explained. Indeed, from the crystal structures of different enzymes, the BRCT domain appears as a flexible unit, with a structure that cannot to be properly resolved, and it is apparently not essential for the adenylation step [51,55,63]. Indeed, NDL proteins not including BRCT in their modular structures are still able to perform their ligation reaction, albeit at a lower rate [62–64]. However, DNA binding was shown to be tighter for an enzyme with a BRCT domain compared with one not containing it [64]. Therefore, the BRCT domain could stabilize the DNA binding on NDLs, even though it does not interact directly with DNA. Another possible function for the BRCT domain could be as a signal transducer of the DNA-damage response; indeed, other well-characterized proteins use this domain for protein-protein interactions to transmit signalling responses [51].

Overall, the modular structure of NDL allows it to catalyse the ligation reaction by coordinating considerable movements of the different domains. It is now clear that similar large-scale conformation movements of the enzymes take place for ligation reactions performed by the majority of NDLs and ADLs.

## ASSAYS OF DNA JOINING BY DNA LIGASES

The reaction catalysed by DNA ligases leads to changes in the ends or backbone structure of DNA and such changes can be detected using a range of biochemical and biophysical assays [65]. Here we briefly review assays that are particularly useful in analysing the influence of small molecules on the activity of DNA ligases.

Gel electrophoresis is a convenient method for detecting the sizes of molecules, and it has been widely used among the primary biochemical studies of DNA ligases that are reviewed here (e.g. see [64,66,67]). However, gel electrophoresis is rather time-consuming and laborious and, thus, is not ideal for drug discovery purposes. Therefore, to facilitate biochemical analyses of DNA ligases and development of inhibitors against them, attention has focused on development of alternative assays to monitor DNA joining activities. Some of these alternatives have been specifically developed to offer increased throughput of assays [68–70], different time resolution or are more cost-effective. In some cases the developments take advantage of novel forms of relevant molecules, such as a fluorescent derivative of β-NAD^+^ that can be processed by different β-NAD^+^-consuming enzymes, including NDLs [71].

A wide range of biophysical chemistry approaches have been used to detect the joining of DNA strand breaks. Those applied to NDLs or ADLs from bacteria or archaea include use of fluorescently-labelled probes [66,70,72,73], FRET [69,74–77], fluorescence quenching and molecular beacon-based approaches [78–81], electrochemical methods [67,79,81–84], a nanoparticle-based sensor [85] and surface plasmon resonance [86]. Biochemical analyses of the ligation reaction are facilitated by assays that do not require ‘labelling’ of the nucleic acid, such as label-free electrochemical approaches using mercury-based electrodes and nicked plasmid DNA substrates [67]. Combinations of techniques can also be powerful, as observed with the electrochemistry alternative that uses a digoxigenin-labelled probe and detects ligation via a ‘double-surface’ enzyme-linked electrochemical assay [84]. Such techniques are fast and inherently versatile due to their easy incorporation of synthetic oligonucleotide substrates, allowing the DNA breaks to be situated in diverse sequence contexts and/or structure perturbations.

As highlighted in many of these assays, oligonucleotide templates that are able to form stem-loop structures are popular since they can be immobilized on different surfaces through biotin–streptavidin interactions or direct linkage to gold surfaces. If the other end of the stem-loop contains a label, then the absence or presence of the label can be used to detect alteration to the backbone structure of DNA. By inclusion of a nick in DNA stem-loop structures, then retention of the signal indicates DNA ligation and such approaches have been monitored using fluorescence [66,78,79] or electrochemical [81,82] techniques. Interestingly, use of appropriate substrates in these assays allows study of the combined action of nucleases and ligases [66], which allows extension of these assays to assess links between DNA ligases and other proteins involved in nucleic acid metabolism, as observed in DNA repair processes.

## INHIBITORS AND NON-NATURAL SUBSTRATES OF DNA LIGASES

Detailed characterization of DNA ligases at a biochemical and molecular level has generated therapeutic interest, with the essential NDLs of bacteria being a suggested target for novel antibiotics candidates [2,7,8,11,12]. The starting point for many studies that aim to inhibit such enzymes is to assess the effects of modified derivatives of the co-factors, either β-NAD^+^ or ATP in the case of DNA ligases. In assessing such compounds, it is important to recognize that they may result in non-natural substrate activity and, thus, may not inhibit the activity of the enzyme [71,87]. Therefore, it is often necessary to test the effects of many different types of modifications to the co-factors. In the case of DNA ligases, modified co-factors have produced a range of effects and it is not straightforward to predict their effects, as we discuss below. Furthermore, an influence on the enzyme activity does not necessarily require major alterations to the chemical groups of the co-factor, as demonstrated recently for T4 DNA ligase exhibiting stereo-selective properties towards modified ATP compounds [88].

The first DNA ligase inhibitors that looked promising for drug discovery purposes were discovered by screening libraries of classes of compounds, such as alkaloids, flavonoids, pyridochromanones, quinoline and quinacrine derivatives [8,89–91]. These compounds were then refined to generate analogues that had improved activity, selectivity, solubility and other factors that make the compounds more ‘druggable’ [53,74–76,92–98]. Importantly, some of these studies also highlighted the challenges associated with developing such compounds as antimicrobial drugs [96].

**Figure 3:**
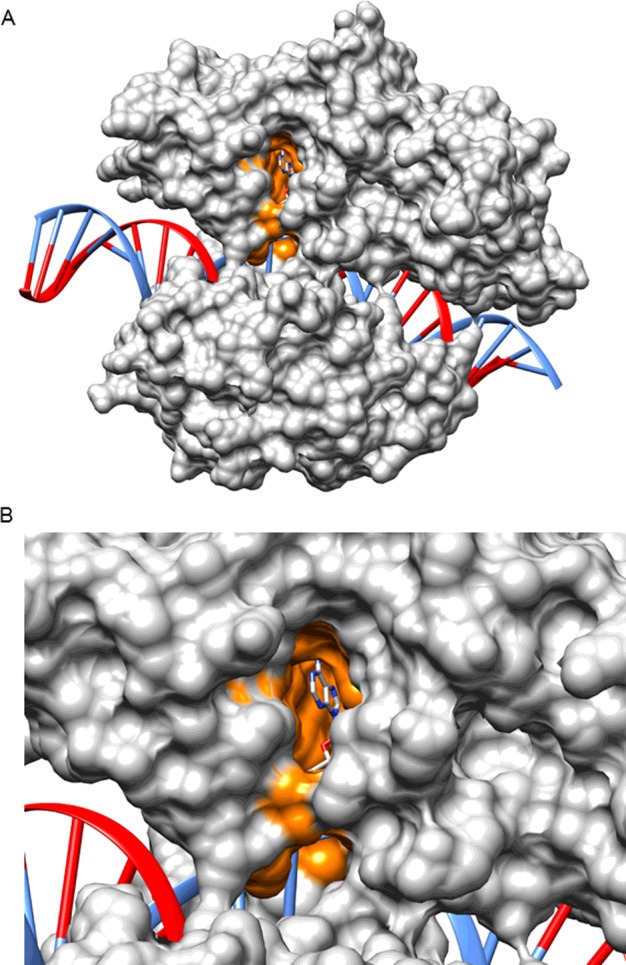
NAD^+^-dependent DNA ligases from bacteria contain a conserved hydrophobic tunnel All NAD^+^-dependent DNA ligases from bacteria contain a hydrophobic tunnel (shaded in orange). The structure is of *E. coli* LigA bound to adenylated DNA from PDB 2OWO [55] and is visualized by UCSF Chimera. The image in (**A**) shows the full structure, with image (**B**) zooming in to give a clearer view of the location of the co-factor in the hydrophobic tunnel. Note that the C-2 of the adenine ring of β-NAD^+^ points in the direction of the hydrophobic tunnel.

**Figure 4:**
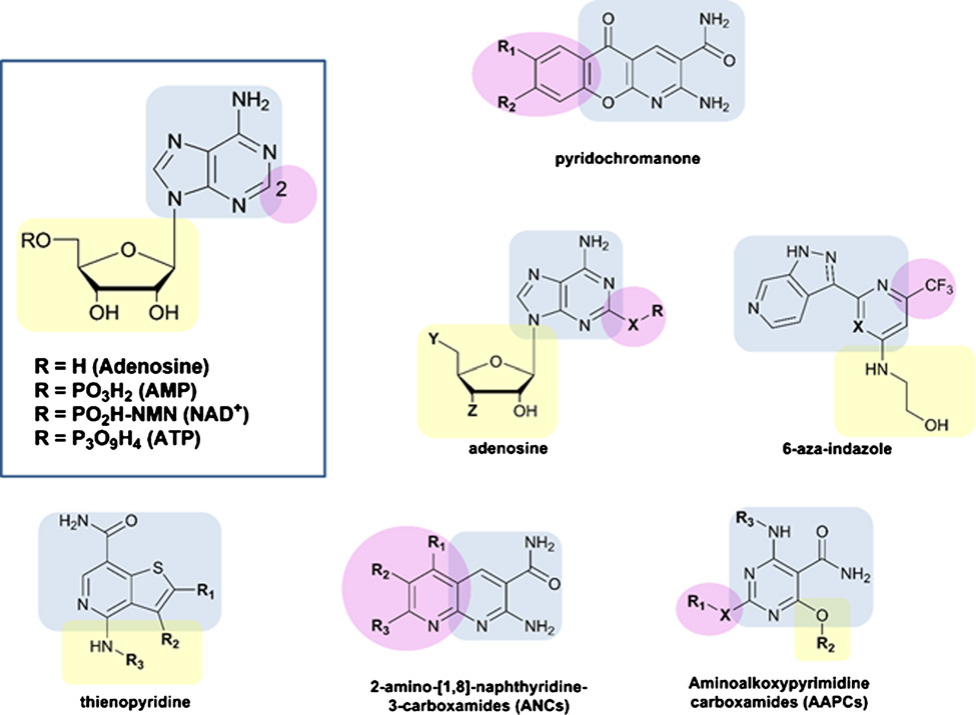
Classes of NAD^+^-dependent DNA ligase inhibitors and their position characteristics compared with the natural substrate The blue box identifies the part of the molecule that binds in the position of the adenine nucleobase; the purple box identifies the part of the molecule that sits within the hydrophobic tunnel of NAD^+^-dependent DNA ligases; the yellow box identifies the sugar moiety binding site.

As described above, a variety of high-resolution structures of NDLs have been resolved in the last decade, providing a better understanding of the β-NAD^+^ binding site [7,55]. The substantial conformational changes of the enzymes that occur during the reaction–and the conformational flexibility of the enzymes in general–makes rational design of inhibitors challenging. By their very nature, structures from crystallographic studies capture only a particular snapshot of the enzyme, which may or may not be relevant for inhibitor binding. Several studies have undertaken basic modelling of the structures of NDLs and virtual screening of molecules that they interact with [8,53,55,61,63,87,91,98–100]. However, it is clear that detailed molecular dynamics studies of NDLs would assist with structure-based design of inhibitors. Information derived from the combination of different structures has highlighted some useful approaches, particularly in relation to the challenges and potential advantages of targeting the co-factor binding site [3–5,7,15]. In particular, identification of the existence of a hydrophobic tunnel (Figure 3) in proximity to N-1, C-2 and N-3 of the adenine ring [7] has opened the opportunity for the development of inhibitors that can fit into this tunnel (Figure 4). Moreover, the tunnel is not present in ADLs, therefore potentially allowing for the generation of inhibitors that are more specific towards NDLs.

Building on previous studies [52], crystallographic structures were obtained for known inhibitors in complex with *E. faecalis* LigA (PDB accession numbers 3BA8, 3BA9, 3BAA, 3BAB; Pinko, Borchardt, Nikulin, Su, unpublished data). These structures showed the inhibitors fitting into the hydrophobic tunnel. Intuitively, adenosine-based compounds substituted on C-2 of the adenine ring fit perfectly into the active site positioning the substituent into the tunnel; consequently, they have become a prominent class of inhibitors (Table 2), extensively studied and optimized to meet optimal drug criteria [92,95]. The scaffold similarity to β-NAD^+^ and the lack of interference of substrate activity make adenosine derivatives very promising inhibitors, with IC_50_ in the low micromolar range.

**Table 2:** IC_50_ values for nucleoside-based inhibitors of NAD^+^-dependent DNA ligases from bacteria *Spn: Streptococcus pneumoniae*; *Sau: Staphylococcus aureus*; *Hin: Haemophilus influenza; Ec: Escherichia coli*; *Mpn: Mycoplasma pneumoniae*. n.d.: not determined. a Details from [75]; ^b^ details from [53]; ^c^ details from [76]; ^d^ details from [74]; e details from [94]. 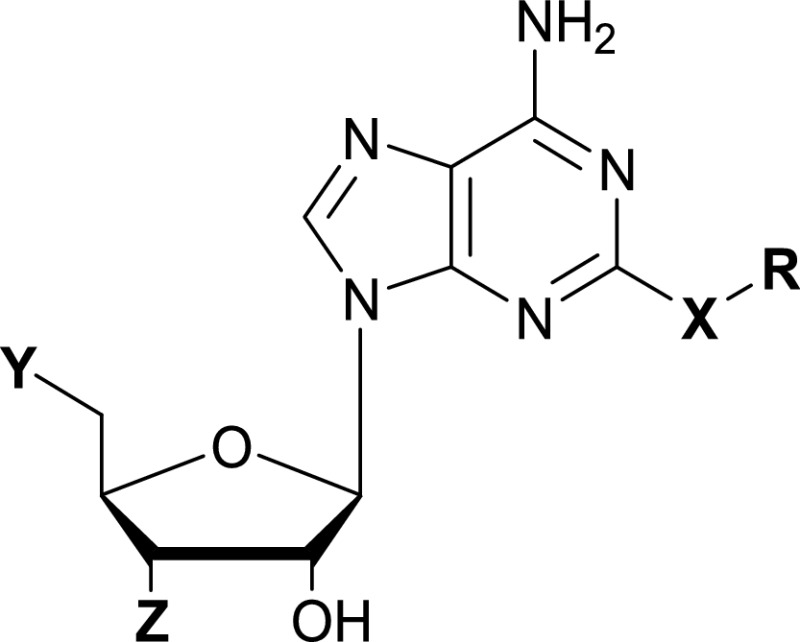

cmpd	X	Y	Z	R	IC_50_ Spn [μM]	IC_50_ Sau [μM]	IC_50_ Hin [μM]	IC_50_ Spn L75F [μM]	IC_50_ Ec [μM]	IC_50_ Mpn [μM]
1^a,c,d^	S	OH	OH	*n*-Butyl	0.14	0.08	0.51	10	1.27	0.07
2a	S	H	OH	*n*-Butyl	0.13	0.17	n.d.	n.d.	n.d.	n.d.
3a	S	OH	OH	*c*-Pentyl	0.44	0.38	n.d.	n.d.	n.d.	n.d.
4^a,b,c^	O	OH	OH	*c*-Pentyl	0.15–0.21	0.43	0.51	30–33	n.d.	n.d.
5a	O	F	OH	*c*-Pentyl	0.12	0.12	n.d.	n.d.	n.d.	n.d.
6^a,d^	O	H	OH	*c*-Pentyl	0.08	0.12	0.11	n.d.	0.37	0.02
7a	O	H	Cl	*c*-Pentyl	0.04	0.04	n.d.	n.d.	n.d.	n.d.
8a	O	F	N3	*c*-Pentyl	0.07	0.09	n.d.	n.d.	n.d.	n.d.
9a	O	OH	Cl	*c*-Pentyl	0.08	0.11	n.d.	n.d.	n.d.	n.d.
10a	O	H	dichlorobenzyl	*c*-Pentyl	<0.01	0.13	n.d.	n.d.	n.d.	n.d.
11^a,d^	O	F	OH	Cyclobutyl methyl	0.05	0.06	0.08	n.d.	0.16	0.02
12^a,c,d^	O	H	OH	(*trans*-4-Methylcyclohexyl)	0.05	0.16	0.11	12	0.32	<0.01
13^a,d^	O	H	OH	Spiro[2.2]pent-1-ylmethyl	0.04	0.06	0.08	n.d.	0.18	0.06
14a	O	H	Cl	Spiro[2.2]pent-1-ylmethyl	0.02	0.03	n.d.	n.d.	n.d.	n.d.
15a	O	H	OH	Bicyclo[3.1.0]hex-3-yl	0.06	0.13	n.d.	n.d.	n.d.	n.d.
16a	O	F	OH	(1-Hydroxymethyl) cyclopropyl methyl	0.40	0.37	n.d.	n.d.	n.d.	n.d.
17a	O	H	OH	Phenyl	2.67	0.14	n.d.	n.d.	n.d.	n.d.
18a	O	F	OH	1,3-Thiazol-2-ylmethyl	0.87	0.91	n.d.	n.d.	n.d.	n.d.
19e	O	H	OH	Decalin	0.04	n.d.	0.26	n.d.	n.d.	n.d.
20e	O	OH	OH	Decalin	0.08	n.d.	0.40	n.d.	n.d.	n.d.
21e	O	H	OH	Adamantane methyl	0.20	n.d.	1.7	n.d.	n.d.	n.d.

Other potential inhibitors that have been assessed include 6-azaindazoles, 2-amino-[1,8]-naphthyridine-3-carboxamides (ANCs) and aminoalkoxypyrimidine carboxamide (AAPCs) (Figure 4). These compounds were modelled and synthesized to resemble the orientation of adenine, ribose and C-2 substituents into the active site with comparable inhibitory activity. Phosphorylated derivatives of adenosine, such as AMP, cAMP, ADP and ATP substituted on C-2 of the adenine ring have also been tested as inhibitors against NDLs with some promising results (Table 3).

**Table 3:** IC_50_ values for nucleotide/dinucleotide-based inhibitors of NAD^+^-dependent DNA ligases from bacteria *c*AMPS: *cyclic* adenosine monophosphothioate; *Ec*: *Escherichia coli*; *Mt*: *Mycobacterium tuberculosis*. n.d.: not determined. a Details from [87]; ^b^ details from [68]. 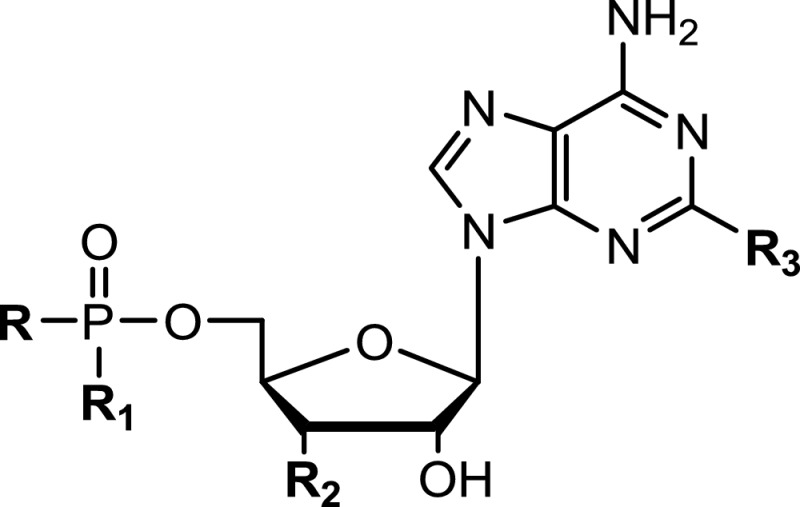

cmpd	R	R_1_	R_2_	R_3_	Scaffold	IC_50_ Ec [μM]	IC_50_ Mt [μM]
22a	OH	OH	OH	Lodo	AMP	120 ± 23	16 ± 8
23a	NMN	OH	OH	Lodo	NAD	138 ± 18	>200
24a	NMN	OH	OH	Phenyl	NAD	n.d.	73 ± 15
25^b^	P_2_O_7_H_3_	OH	OH	*S*-Methyl	ATP	1.1	n.d.
26^b^	PO_4_H_2_	OH	OH	*S*-Methyl	ADP	4.1	n.d.
27^b^	S	R_1_=R_2_	O	*S*-Methyl	*c*AMPS	1.5 (S) 120 (R)	n.d.

Despite this progress, there have been relatively few reports of β-NAD^+^ derivatives as inhibitors of NDLs (Table 3). The reasons for this include the difficult synthesis of β-NAD^+^ derivatives, their relatively limited and modest stability, and their flexible/multifunctional structure, which makes β-NAD^+^ binding quite unspecific. Such modified derivatives of β-NAD^+^ may also result in non-natural substrate activity, though this property can be exploited to provide useful insight into the catalytic mechanisms of the enzymes [71,87].

Recent studies have assessed the biological activity of the β-NAD^+^ derivatives and some of their corresponding AMP and AMP-morpholidate derivatives. For example, using EcLigA and MtLigA, the enzymes from *E. coli* and *M. tuberculosis* that are among the best characterized NDLs, compounds modified in position 2, 6 or 8 of the adenine ring were tested as inhibitors and/or non-natural substrates [87]. The different substitution patterns on derivatives of β-NAD^+^ and AMP had a pronounced effect on the biochemical activity of EcLigA and MtLigA. The 6-substituted derivatives were neither co-substrates (in the case of the β-NAD^+^ derivatives) nor inhibitors (in the case of both β-NAD^+^ and AMP derivatives) [87]. These results suggest that the presence of an additional substituent in position 6 is not tolerated by either EcLigA or MtLigA, and is detrimental for co-substrate binding. None of the 8-substituted β-NAD^+^ derivatives were utilized as co-substrates by EcLigA either, but two were weak inhibitors [87]. This pattern of behaviour suggests that 8-substituted β-NAD^+^ derivatives can bind at the active site of NDLs, but do so in a non-productive orientation. Due to the proximity of the protein backbone and position 8 of the adenine ring, there is not enough space within the active site to accommodate an additional substituent at this position without changing the original position of the adenine. Thus, the binding of 8-substituted β-NAD^+^ derivatives at the active site probably occurs in a different orientation from that of natural β-NAD^+^ and, consequently, precludes establishment of key interactions within the active site and, therefore, their co-substrate activity [87]. By contrast, co-substrate and/or inhibitory activities were observed in the series of 2-substituted derivatives highlighting that an additional substituent at the 2-position of β-NAD^+^/AMP can be accommodated by NDLs and may be involved in direct interactions with the target DNA ligase [87]. This hypothesis is supported by results from molecular docking experiments taking advantage of the structure of EcLigA (PDB: 2OWO [55]), which suggest that 2-substituted AMP/β-NAD^+^ derivatives can be accommodated at the active site in an orientation that is nearly identical with that of natural AMP/β-NAD^+^ [87]. In this position, the adenine ring of the compounds maintains all key interactions with active site residues, whereas the substituent in position 2 fits into the hydrophobic tunnel adjacent to the adenine binding site (Figure 3). However, different substituents may interact differently with the residues in the tunnel; thus, although the 2-iodo substituent may electrostatically interact with Lys-290 at the entrance of the hydrophobic tunnel, a phenyl substituent does not appear to make any specific contacts with residues in the tunnel, explaining the superior inhibitory activity of 2-iodo derivatives against 2-phenyl derivatives toward EcLigA (Table 3).

**Figure 5:**
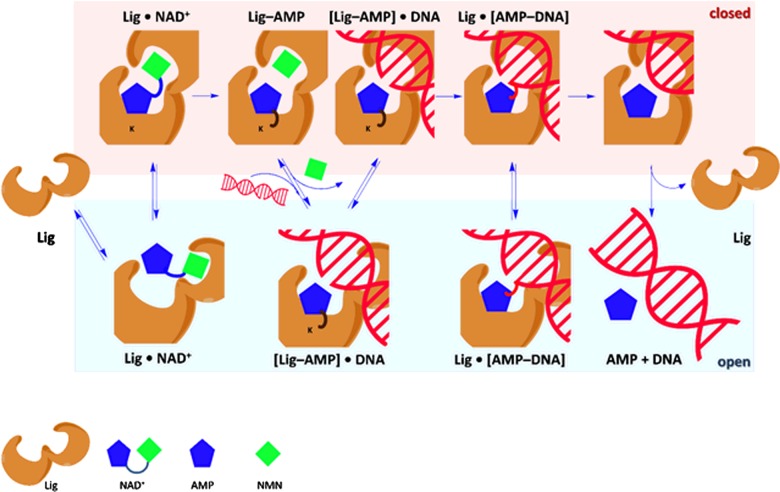
Reaction mechanism for DNA ligation by NAD^+^-dependent DNA ligases The enzyme uses a ping-pong mechanism, with β-NAD^+^ leading to an adenylated intermediate (Lig-AMP) at a conserved nucleophilic lysine residue. The N-terminal adenylation domain of DNA ligases include binding sites for the NMN (subdomain 1a) and AMP (subdomain 1b) moieties of β-NAD^+^. The binding of the β-NAD^+^ co-factor triggers the closure of subdomain 1a on to subdomain 1b, and the transition from an ‘open’ to a ‘closed’ conformation, which is critical for DNA ligase activity. In the closed conformation, the adenine of β-NAD^+^ is bound tightly to the active site by several key interactions with conserved residues. These interactions hold the adenine ring in place although the conformation of the AMP moiety changes from *syn* in non-covalently bound β-NAD^+^ to anti in the adenylate-ligase intermediate, and back to *syn* in the adenylated-DNA intermediate. Progress from one ‘open’ intermediate to the next can only occur by passing through a ‘closed’ intermediate, meaning that multiple, large-scale changes take place during the overall reaction; see text for further details. Similar large-scale conformational changes occur during ligation by ATP-dependent DNA ligases, but the specific details vary for each version of those enzymes.

Moreover, the availability of the two pairs of AMP/β-NAD^+^ derivatives with the same 2-substituent (2-iodo or 2-phenyl) enables a detailed assessment of the role of the NMN fragment for DNA ligase inhibition by base-modified adenine nucleotides. The presence of the complete β-NAD^+^ scaffold, although not an absolute prerequisite for inhibitory activity, altered the profile of inhibition, leading to synergistic effects with β-NAD^+^ or overcoming the time-dependence of inhibition. Interestingly, this inhibitory profile was again dependent on the nature of the 2-substituent suggesting that it has an important role for orienting the NAD^+^ scaffold in the active site, and consequently for subdomain closure [87]. In addition, a different general trend for inhibition in the NAD^+^ derivatives against EcLigA and MtLigA highlight that subtle differences in the shape of the hydrophobic tunnel between EcLigA and MtLigA critically influence the binding of different 2-substituents and, consequently, the orientation of 2-substituted β-NAD^+^ derivatives in the active site of the respective DNA ligase. The results described for 2-substituted derivatives of β-NAD^+^ [87] suggest that their effects are influenced by the large conformational changes required for full activity of NDLs, highlighting the involvement of two distinct nucleotide binding sites (see Figure 5, below). Additional, detailed studies are required to identify which particular steps in the reaction mechanism are inhibited by the variously modified derivatives of β-NAD^+^ and to assess whether any of them have any effects on bacteria.

Recently, the potential usefulness of adenosine-based inhibitors carrying a substituent on C-2 of the adenine ring has been questioned with the discovery of a ligase mutant, L75F, which restricts the tunnel with >10-fold loss in antibacterial activity and >100-fold loss in target binding [74,76]. A more recent class of inhibitors based on a thienopyridine scaffold (Figure 4) avoids the presence of substituents fitting into the tunnel and resembles only the orientation of adenine and ribose within the binding site.

## MECHANISM OF DNA LIGATION BY NAD^+^-DEPENDENT DNA LIGASES

Biochemical studies of DNA ligases began with discovery of the enzymes in the 1960s. The most intensively studied NDL is EcLigA from *E. coli*, which is unsurprising since it was the first bacterial DNA ligase to be characterized biochemically [1–3,13]. Studies of EcLigA continue to give useful insights into the reaction mechanism of NDLs, as observed with a recent kinetic analysis that examined the way in which base-pairing at the nick in the DNA influences the activity of the enzyme [101].

As already highlighted, NDLs follow a ping-pong mechanism (Figure 5), with β-NAD^+^ leading to an adenylated intermediate (E-AMP) at a conserved nucleophilic lysine residue (Lys-115 in EcLigA). Throughout these reaction steps, both the DNA ligase and the bound β-NAD^+^ co-factor undergo significant conformational changes. The two subdomains 1a and 1b of the N-terminal adenylation domain of DNA ligases include binding sites for, respectively, the NMN (subdomain 1a) and AMP (subdomain 1b) moieties of β-NAD^+^. The binding of the β-NAD^+^ co-factor triggers the closure of subdomain 1a on to subdomain 1b, and the transition from an ‘open’ to a ‘closed’ conformation (Figure 5), which is critical for DNA ligase activity [52,57]. Note that progress from one ‘open’ intermediate to the next can only occur by passing through a ‘closed’ intermediate, meaning that multiple, large-scale changes take place during the overall reaction. Studies with *H. influenzae* LigA suggest that the initial recognition of β-NAD^+^ occurs at the NMN binding site on subdomain 1a, providing an explanation for the selectivity of bacterial DNA ligases for β-NAD^+^ over ATP [57]. In the closed conformation, the adenine of β-NAD^+^ is bound tightly to the active site by several key interactions with conserved residues, such as Lys-290 (H-bonding to N1), Glu-113 (H-bonding to N6) and Tyr-225 (π–π stacking) in EcLigA; in particular, the interaction with Lys-290 is critical for enzymatic activity [55]. Taken together, these interactions hold the adenine ring in place, although the conformation of the AMP moiety changes, through rotation of the ribose about the N-glycosidic bond, from *syn* in non-covalently bound β-NAD^+^, to anti in the adenylate-ligase intermediate, and back to *syn* in the adenylated-DNA intermediate [55].

## CONCLUSIONS

DNA ligases are essential enzymes for all cells because they seal breaks in DNA backbones and, thus, ensure that its structural integrity is maintained. Recognition that the essential DNA ligases in bacteria use a different co-factor (β-NAD^+^) compared with the ATP co-factor that is used by the eukaryotic enzymes raised hopes that they could be targeted by novel antibiotics. Until recently, progress in this aim has been hampered due to the difficult synthesis of β-NAD^+^ derivatives and their relatively limited stability. However, the complex molecular structure of β-NAD^+^ affords multiple opportunities for chemical modification and several recent studies have synthesized promising novel derivatives.

The biological activity of these compounds have been evaluated as inhibitors for drug discovery and such modified derivatives of β-NAD^+^ also result in non-natural substrate activity that can be exploited to provide useful insight into the catalytic mechanisms of DNA ligases. Summarizing recent results, it is clear that aryl/heteroaryl substitutions on different positions of the adenine ring generate β-NAD^+^ derivatives with biological activities toward β-NAD^+^-consuming enzymes. The preferential action of aryl/heteroaryl adenine-modified β-NAD^+^ derivatives as inhibitors and/or non-natural substrates toward specific β-NAD^+^-consuming enzymes offers the prospect of their use for *in vitro*/*in vivo* studies. Such a step forward in their biological evaluation will require consideration of their activity and stability within cells, such as their ability to resist degradation by ecto β-NAD^+^-consuming enzymes or intracellular nucleases. Therefore, chemical modifications on the β-NAD^+^ scaffold or alternative methods of delivery will need to be developed with a focus on the use for which they are designed. However, the validity of the use of such compounds as biochemical probes is already a useful place to widen the knowledge of fundamental biological processes involving β-NAD^+^-consuming enzymes.

Biochemical and structural studies have made significant progress in identifying compounds that inhibit NDLs. However, the prospects of developing such compounds as antimicrobial drugs remains challenging, particularly since it is likely that effective drugs will have to inhibit NDLs very efficiently, with very good discrimination against ADLs [21,96]. Recent comparative analysis of co-factor and inhibition experiments raises the possibility that useful inhibition may be obtained due to targeting of the significant conformational changes that occur within DNA ligases during their catalytic cycle. Importantly, such findings may provide a promising starting point for the rational design of new classes of inhibitors against bacterial DNA ligases. It is also notable that most studies of potential inhibitors have assessed the effects of compounds that bind reversibly to DNA ligases. Compounds that bind to NDLs through covalent binding could offer novel solutions to the need to inhibit the enzymes very efficiently in the cell. Detailed molecular dynamics studies are likely to offer valuable insights into useful avenues to explore in the development of improved inhibitors of NDLs.

In summary, although progress to identify useful compounds that target DNA ligases has been rather frustrating, the recent development of modified derivatives of nucleotides offers encouragement for such studies. The continued combination of structural, biochemical and biophysical techniques will be useful in targeting these essential cellular enzymes.

## Abbreviations

β-NAD^+^: β-nicotinamide adenine dinucleotide
β-NMN: β-nicotinamide mononucleotide
ADL: ATP-dependent DNA ligase
ATP: adenosine-5′-triphosphate
BRCT: breast cancer carboxy-terminal
HhH: helix–hairpin–helix motif
NDL: NAD^+^-dependent DNA ligase
NHEJ: non-homologous end-joining
NTase: nucleotidyltransferase domain
OB site: oligonucleotide binding site

## References

[1] Timson D.J., Singleton M.R., Wigley D.B. (2000). DNA ligases in the repair and replication of DNA. Mutat. Res..

[2] Wilkinson A., Day J., Bowater R. (2001). Bacterial DNA ligases. Mol. Microbiol..

[3] Shuman S., Lima C.D. (2004). The polynucleotide ligase and RNA capping enzyme superfamily of covalent nucleotidyltransferases. Curr. Opin. Struct. Biol..

[4] Tomkinson A.E., Vijayakumar S., Pascal J.M., Ellenberger T. (2006). DNA ligases: structure, reaction mechanism, and function. Chem. Rev..

[5] Pascal J.M. (2008). DNA and RNA ligases: structural variations and shared mechanisms. Curr. Opin. Struct. Biol..

[6] Ellenberger T., Tomkinson A.E. (2008). Eukaryotic DNA ligases: structural and functional insights. Annu. Rev. Biochem..

[7] Shuman S. (2009). DNA ligases: progress and prospects. J. Biol. Chem..

[8] Dwivedi N., Dube D., Pandey J., Singh B., Kukshal V., Ramachandran R. (2008). NAD(^+^)-dependent DNA ligase: a novel target waiting for the right inhibitor. Med. Res. Rev.

[9] Martin I.V., MacNeill S.A. (2002). ATP-dependent DNA ligases. Genome Biol..

[10] Williamson A., Hjerde E., Kahlke T. (2016). Analysis of the distribution and evolution of the ATP-dependent DNA ligases of bacteria delineates a distinct phylogenetic group ‘Lig E'. Mol. Microbiol..

[11] Pankiewicz K.W., Petrelli R., Singh R., Felczak K. (2015). Nicotinamide adenine dinucleotide based therapeutics, update. Curr. Med. Chem..

[12] Hale M.R., Brassington C., Carcanague D., Embrey K., Eyermann C.J., Giacobbe R.A. (2015). From fragments to leads: novel bacterial NAD^+^-dependent DNA ligase inhibitors. Tetrahedron Lett..

[13] Lehman I.R. (1974). DNA ligase: structure, mechanism, and function. Science.

[14] Chambers C.R., Patrick W.M. (2015). Archaeal nucleic acid ligases and their potential in biotechnology. Archaea.

[15] Tanabe M., Ishino Y., Nishida H. (2015). From structure-function analyses to protein engineering for practical applications of DNA ligase. Archaea.

[16] Finn R.D., Coggill P., Eberhardt R.Y., Eddy S.R., Mistry J., Mitchell A.L. (2016). The Pfam protein families database: towards a more sustainable future. Nucleic Acids Res..

[17] Williams T.A., Foster P.G., Cox C.J., Embley T.M. (2013). An archaeal origin of eukaryotes supports only two primary domains of life. Nature.

[18] Embley T.M., Williams T.A. (2015). Evolution: steps on the road to eukaryotes. Nature.

[19] Spang A., Saw J.H., Jorgensen S.L., Zaremba-Niedzwiedzka K., Martijn J., Lind A.E. (2015). Complex archaea that bridge the gap between prokaryotes and eukaryotes. Nature.

[20] Tomkinson A.E., Sallmyr A. (2013). Structure and function of the DNA ligases encoded by the mammalian LIG3 gene. Gene.

[21] Korycka-Machala M., Rychta E., Brzostek A., Sayer H., Rumijowska-Galewicz A., Bowater R. (2007). Evaluation of NAD^+^-dependent DNA ligase of mycobacteria as a potential target for antibiotics. Antimicrobial. Agents Chemother..

[22] Gong C., Bongiorno P., Martins A., Stephanou N.C., Zhu H., Shuman S. (2005). Mechanism of nonhomologous end-joining in mycobacteria: a low-fidelity repair system driven by Ku, ligase D and ligase C. Nat. Struct. Mol. Biol..

[23] Bowater R.P., Doherty A.J. (2006). Making ends meet: repairing breaks in bacterial DNA by non-homologous end-joining. PLoS Genetics.

[24] Korycka-Machala M., Brzostek A., Rozalska S., Rumijowska-Galewicz A., Dziedzic R., Bowater R. (2006). Distinct DNA repair pathways involving RecA and nonhomologous end joining in *Mycobacterium*
*smegmatis*. FEMS Microbiol. Letts..

[25] Pitcher R.S., Brissett N.C., Doherty A.J. (2007). Nonhomologous end-joining in bacteria: a microbial perspective. Annu. Rev. Microbiol..

[26] Shuman S., Glickman M.S. (2007). Bacterial DNA repair by non-homologous end joining. Nat. Rev. Microbiol..

[27] Pascal J.M., Tsodikov O.V., Hura G.L., Song W., Cotner E.A., Classen S. (2006). A flexible interface between DNA ligase and PCNA supports conformational switching and efficient ligation of DNA. Mol. Cell.

[28] Nishida H., Kiyonari S., Ishino Y., Morikawa K. (2006). The closed structure of an archaeal DNA ligase from *Pyrococcus*
*furiosus*. J. Mol. Biol..

[29] Kim D.J., Kim O., Kim H.W., Kim H.S., Lee S.J., Suh S.W. (2009). ATP-dependent DNA ligase from *Archaeoglobus*
*fulgidus* displays a tightly closed conformation. Acta Crystallogr. Sect. F Struct. Biol. Cryst. Commun..

[30] Supangat S., An Y.J., Sun Y., Kwon S.T., Cha S.S. (2010). Purification, crystallization and preliminary crystallographic analysis of a multiple cofactor-dependent DNA ligase from *Sulfophobococcus*
*zilligii*. Acta Crystallogr. Sect. F Struct. Biol. Cryst. Commun..

[31] Petrova T., Bezsudnova E.Y., Boyko K.M., Mardanov A.V., Polyakov K.M., Volkov V.V. (2012). ATP-dependent DNA ligase from *Thermococcus*
*sp*. 1519 displays a new arrangement of the OB-fold domain. Acta Crystallogr. Sect. F Struct. Biol. Cryst. Commun..

[32] Petrova T.E., Bezsudnova E.Y., Dorokhov B.D., Slutskaya E.S., Polyakov K.M., Dorovatovskiy P.V. (2012). Expression, purification, crystallization and preliminary crystallographic analysis of a thermostable DNA ligase from the archaeon *Thermococcus*
*sibiricus*. Acta Crystallogr. Sect. F Struct. Biol. Cryst. Commun..

[33] Tanabe M., Ishino S., Ishino Y., Nishida H. (2014). Mutations of Asp540 and the domain-connecting residues synergistically enhance *Pyrococcus*
*furiosus* DNA ligase activity. FEBS Lett..

[34] Akey D., Martins A., Aniukwu J., Glickman M.S., Shuman S., Berger J.M. (2006). Crystal structure and nonhomologous end-joining function of the ligase component of mycobacterium DNA ligase D. J. Biol. Chem..

[35] Williamson A., Rothweiler U., Leiros H.K. (2014). Enzyme-adenylate structure of a bacterial ATP-dependent DNA ligase with a minimized DNA-binding surface. Acta Crystallogr. D Biol. Crystallogr..

[36] Stephanou N.C., Gao F., Bongiorno P., Ehrt S., Schnappinger D., Shuman S. (2007). Mycobacterial nonhomologous end joining mediates mutagenic repair of chromosomal double-strand DNA breaks. J. Bacteriol..

[37] Zhu H., Shuman S. (2007). Characterization of *Agrobacterium*
*tumefaciens* DNA ligases C and D. Nucl. Acids Res..

[38] Aniukwu J., Glickman M.S., Shuman S. (2008). The pathways and outcomes of mycobacterial NHEJ depend on the structure of the broken DNA ends. Genes Dev..

[39] Zhu H., Shuman S. (2010). Gap filling activities of Pseudomonas DNA ligase D (LigD) polymerase and functional interactions of LigD with the DNA end-binding Ku protein. J. Biol. Chem..

[40] Gupta R., Barkan D., Redelman-Sidi G., Shuman S., Glickman M.S. (2011). Mycobacteria exploit three genetically distinct DNA double-strand break repair pathways. Mol. Microbiol..

[41] Zhu H., Shuman S. (2005). Novel 3′ ribonuclease and 3′ phosphatase activities of the bacterial NHEJ protein, DNA ligase D. J. Biol. Chem..

[42] Zhu H., Shuman S. (2006). Substrate specificity and structure-function analysis of the 3′-phosphoesterase component of the bacterial NHEJ protein, DNA ligase D. J. Biol. Chem..

[43] Nair P.A., Smith P., Shuman S. (2010). Structure of bacterial LigD 3′-phosphoesterase unveils a DNA repair superfamily. Proc. Natl. Acad. Sci. U.S.A..

[44] Smith P., Nair P.A., Das U., Zhu H., Shuman S. (2011). Structures and activities of archaeal members of the LigD 3′-phosphoesterase DNA repair enzyme superfamily. Nucleic Acids Res..

[45] Bartlett E.J., Brissett N.C., Doherty A.J. (2013). Ribonucleolytic resection is required for repair of strand displaced nonhomologous end-joining intermediates. Proc. Natl. Acad. Sci. U.S.A..

[46] Borrel G., Harris H.M., Tottey W., Mihajlovski A., Parisot N., Peyretaillade E. (2012). Genome sequence of “Candidatus Methanomethylophilus alvus” Mx1201, a methanogenic archaeon from the human gut belonging to a seventh order of methanogens. J. Bacteriol..

[47] Zhao A., Gray F.C., MacNeill S.A. (2006). ATP- and NAD^+^-dependent DNA ligases share an essential function in the halophilic archaeon *Haloferax*
*volcanii*. Mol. Microbiol..

[48] Giroux X., MacNeill S.A. (2015). Inhibiting NAD+-dependent DNA ligase activity with 2-(cyclopentyloxy)-5′-deoxyadenosine (CPOdA) offers a new tool for DNA replication and repair studies in the model archaeon *Haloferax*
*volcanii*. FEMS Microbiol. Lett..

[49] Poidevin L., MacNeill S.A. (2006). Biochemical characterisation of LigN, an NAD^+^-dependent DNA ligase from the halophilic euryarchaeon *Haloferax*
*volcanii* that displays maximal *in vitro* activity at high salt concentrations. BMC Mol. Biol..

[50] Singleton M.R., Hakansson K., Timson D.J., Wigley D.B. (1999). Structure of the adenylation domain of an NAD^+^-dependent DNA ligase. Structure.

[51] Lee J.Y., Chang C., Song H.K., Moon J., Yang J.K., Kim H.K. (2000). Crystal structure of NAD(^+^)-dependent DNA ligase: modular architecture and functional implications. EMBO J..

[52] Gajiwala K.S., Pinko C. (2004). Structural rearrangement accompanying NAD^+^ synthesis within a bacterial DNA ligase crystal. Structure (Camb).

[53] Murphy-Benenato K., Wang H., McGuire H.M., Davis H.E., Gao N., Prince D.B. (2014). Identification through structure-based methods of a bacterial NAD(^+^)-dependent DNA ligase inhibitor that avoids known resistance mutations. Bioorg. Med. Chem. Lett..

[54] Srivastava S.K., Tripathi R.P., Ramachandran R. (2005). NAD^+^-dependent DNA ligase (Rv3014c) from *Mycobacterium tuberculosis*. Crystal structure of the adenylation domain and identification of novel inhibitors. J. Biol. Chem..

[55] Nandakumar J., Nair P.A., Shuman S. (2007). Last stop on the road to repair: structure of *E. coli* DNA ligase bound to nicked DNA-adenylate. Mol. Cell..

[56] Surivet J.P., Lange R., Hubschwerlen C., Keck W., Specklin J.L., Ritz D. (2012). Structure-guided design, synthesis and biological evaluation of novel DNA ligase inhibitors with *in vitro* and *in vivo* anti-staphylococcal activity. Bioorg. Med. Chem. Lett..

[57] Lahiri S.D., Gu R.F., Gao N., Karantzeni I., Walkup G.K., Mills S.D. (2012). Structure guided understanding of NAD^+^ recognition in bacterial DNA ligases. ACS Chem. Biol..

[58] Howard S., Amin N., Benowitz A.B., Chiarparin E., Cui H., Deng X. (2013). Fragment-based discovery of 6-azaindazoles as inhibitors of bacterial DNA ligase. ACS Med. Chem. Lett..

[59] Wang L.K., Zhu H., Shuman S. (2009). Structure-guided mutational analysis of the nucleotidyltransferase domain of *Escherichia coli* DNA ligase (LigA). J. Biol. Chem..

[60] Zhu H., Shuman S. (2005). Structure-guided mutational analysis of the nucleotidyltransferase domain of *Escherichia coli* NAD^+^-dependent DNA ligase (LigA). J. Biol. Chem..

[61] Srivastava S.K., Dube D., Tewari N., Dwivedi N., Tripathi R.P., Ramachandran R. (2005). *Mycobacterium tuberculosis* NAD^+^-dependent DNA ligase is selectively inhibited by glycosylamines compared with human DNA ligase I. Nucleic Acids Res..

[62] Wang L.K., Nair P.A., Shuman S. (2008). Structure-guided mutational analysis of the OB, HhH, and BRCT domains of *Escherichia coli* DNA ligase. J. Biol. Chem..

[63] Srivastava S.K., Dube D., Kukshal V., Jha A.K., Hajela K., Ramachandran R. (2007). NAD^+^-dependent DNA ligase (Rv3014c) from *Mycobacterium tuberculosis*: novel structure-function relationship and identification of a specific inhibitor. Proteins.

[64] Wilkinson A., Smith A., Bullard D., Lavesa-Curto M., Sayer H., Bonner A. (2005). Analysis of ligation and DNA binding by *Escherichia coli* DNA ligase (LigA). Biochim. Biophys. Acta.

[65] Bowater R.P., Cobb A.M., Pivonkova H., Havran L., Fojta M. (2015). Biophysical and electrochemical studies of protein–nucleic acid interactions. Monatshefte für Chemie.

[66] Scott B.O.S., Lavesa-Curto M., Bullard D.R., Butt J.N., Bowater R.P. (2006). Immobilized DNA hairpins for assay of sequential breaking and joining of DNA backbones. Anal. Biochem..

[67] Vacek J., Cahova K., Palecek E., Bullard D.R., Lavesa-Curto M., Bowater R.P. (2008). Label-free electrochemical monitoring of DNA ligase activity. Anal. Chem..

[68] Miesel L., Kravec C., Xin A.T., McMonagle P., Ma S., Pichardo J. (2007). A high-throughput assay for the adenylation reaction of bacterial DNA ligase. Anal. Biochem..

[69] Shapiro A.B., Eakin A.E., Walkup G.K., Rivin O. (2011). A high-throughput fluorescence resonance energy transfer-based assay for DNA ligase. J. Biomol. Screen.

[70] Lohman G.J., Bauer R.J., Nichols N.M., Mazzola L., Bybee J., Rivizzigno D. (2016). A high-throughput assay for the comprehensive profiling of DNA ligase fidelity. Nucleic Acids Res..

[71] Pergolizzi G., Butt J.N., Bowater R.P., Wagner G.K. (2011). A novel fluorescent probe for NAD-consuming enzymes. Chem. Commun. (Camb).

[72] Ren J., Wang J., Wang J., Wang E. (2014). Inhibition of G-quadruplex assembling by DNA ligation: a versatile and non-covalent labeling strategy for bioanalysis. Biosens. Bioelectron..

[73] Nikiforov T.T., Roman S. (2015). Fluorogenic DNA ligase and base excision repair enzyme assays using substrates labeled with single fluorophores. Anal. Biochem..

[74] Mills S.D., Eakin A.E., Buurman E.T., Newman J.V., Gao N., Huynh H. (2011). Novel bacterial NAD^+^-dependent DNA ligase inhibitors with broad-spectrum activity and antibacterial efficacy *in vivo*. Antimicrob. Agents Chemother..

[75] Stokes S.S., Huynh H., Gowravaram M., Albert R., Cavero-Tomas M., Chen B. (2011). Discovery of bacterial NAD^+^-dependent DNA ligase inhibitors: optimization of antibacterial activity. Bioorg. Med. Chem. Lett..

[76] Jahic H., Liu C.F., Thresher J., Livchak S., Wang H., Ehmann D.E. (2012). The kinetic mechanism of *S.*
*pneumoniae* DNA ligase and inhibition by adenosine-based antibacterial compounds. Biochem. Pharmacol..

[77] Shapiro A.B. (2014). Complete steady-state rate equation for DNA ligase and its use for measuring product kinetic parameters of NAD(^+^)-dependent DNA ligase from *Haemophilus*
*influenzae*. BMC Res. Notes.

[78] Liu L., Tang Z., Wang K., Tan W., Li J., Guo Q. (2005). Using molecular beacon to monitor activity of *E. coli* DNA ligase. Analyst.

[79] Wu Z.S., Jiang J.H., Shen G.L., Yu R.Q. (2007). Highly sensitive DNA detection and point mutation identification: an electrochemical approach based on the combined use of ligase and reverse molecular beacon. Human. Mutation.

[80] Wang H., Li J., Wang Y., Jin J., Yang R., Wang K. (2010). Combination of DNA ligase reaction and gold nanoparticle-quenched fluorescent oligonucleotides: a simple and efficient approach for fluorescent assaying of single-nucleotide polymorphisms. Anal. Chem..

[81] He X.X., Ni X.Q., Wang Y.H., Wang K.M., Jian L.X. (2011). Electrochemical detection of nicotinamide adenine dinucleotide based on molecular beacon-like DNA and *E. coli* DNA ligase. Talanta.

[82] Zauner G., Wang Y., Lavesa-Curto M., Macdonald A., Mayes A.G., Bowater R.P. (2005). Tethered DNA hairpins facilitate electrochemical detection of DNA ligation. Analyst.

[83] Zhang P., Chu X., Xu X.M., Shen G.L., Yu R.Q. (2008). Electrochemical detection of point mutation based on surface ligation reaction and biometallization. Biosens. Bioelectron..

[84] Stejskalova E., Horakova P., Vacek J., Bowater R.P., Fojta M. (2014). Enzyme-linked electrochemical DNA ligation assay using magnetic beads. Anal. Bioanal. Chem..

[85] Pang L.L., Li J.S., Jiang J.H., Le Y., Shen G.L., Yu R.Q. (2007). A novel detection method for DNA point mutation using QCM based on Fe3O4/Au core/shell nanoparticle and DNA ligase reaction. Sens. Actuator. B-Chem..

[86] Luan Q., Xue Y., Yao X., Lu W. (2010). Hairpin DNA probe based surface plasmon resonance biosensor used for the activity assay of *E. coli* DNA ligase. Analyst.

[87] Pergolizzi G., Cominetti M.M., Butt J.N., Field R.A., Bowater R.P., Wagner G.K. (2015). Base-modified NAD and AMP derivatives and their activity against bacterial DNA ligases. Org. Biomol. Chem..

[88] Pawlowska R., Korczynski D., Nawrot B., Stec W.J., Chworos A. (2016). The alpha-thio and/or beta-gamma-hypophosphate analogs of ATP as cofactors of T4 DNA ligase. Bioorg. Chem..

[89] Ciarrocchi G., MacPhee D.G., Deady L.W., Tilley L. (1999). Specific inhibition of the eubacterial DNA ligase by arylamino compounds. Antimicrob. Agents Chemother..

[90] Meier T.I., Yan D., Peery R.B., McAllister K.A., Zook C., Peng S.B. (2008). Identification and characterization of an inhibitor specific to bacterial NAD^+^-dependent DNA ligases. FEBS J..

[91] Tripathi R.P., Pandey J., Kukshal V., Ajay A., Mishra M., Dube D. (2011). Synthesis, *in*
*silico* screening and bioevaluation of dispiro-cycloalkanones as antitubercular and mycobacterial NAD^+^-dependent DNA ligase inhibitors. MedChemComm.

[92] Gu W., Wang T., Maltais F., Ledford B., Kennedy J., Wei Y. (2012). Design, synthesis and biological evaluation of potent NAD^+^-dependent DNA ligase inhibitors as potential antibacterial agents. Part I: aminoalkoxypyrimidine carboxamides. Bioorg. Med. Chem. Lett..

[93] Stokes S.S., Gowravaram M., Huynh H., Lu M., Mullen G.B., Chen B. (2012). Discovery of bacterial NAD(^+^)-dependent DNA ligase inhibitors: improvements in clearance of adenosine series. Bioorg. Med. Chem. Lett..

[94] Buurman E.T., Laganas V.A., Liu C.F., Manchester J.I. (2012). Antimicrobial activity of adenine-based inhibitors of NAD^+^-dependent DNA ligase. ACS Med. Chem. Lett..

[95] Wang T., Duncan L., Gu W., O'Dowd H., Wei Y., Perola E. (2012). Design, synthesis and biological evaluation of potent NAD^+^-dependent DNA ligase inhibitors as potential antibacterial agents. Part 2: 4-amino-pyrido[2,3-d]pyrimidin-5(8H)-ones. Bioorg. Med. Chem. Lett..

[96] Podos S.D., Thanassi J.A., Pucci M.J. (2012). Mechanistic assessment of DNA ligase as an antibacterial target in *Staphylococcus*
*aureus*. Antimicrob. Agents Chemother..

[97] Murphy-Benenato K.E., Gingipalli L., Boriack-Sjodin P.A., Martinez-Botella G., Carcanague D., Eyermann C.J. (2015). Negishi cross-coupling enabled synthesis of novel NAD(^+^)-dependent DNA ligase inhibitors and SAR development. Bioorg. Med. Chem. Lett..

[98] Yadav N., Khanam T., Shukla A., Rai N., Hajela K., Ramachandran R. (2015). Tricyclic dihydrobenzoxazepine and tetracyclic indole derivatives can specifically target bacterial DNA ligases and can distinguish them from human DNA ligase I. Org. Biomol. Chem..

[99] Vijayalakshmi P., Daisy P. (2015). Effective interaction studies for inhibition of DNA ligase protein from *Staphylococcus*
*aureus*. J. Recept. Signal. Transduct. Res..

[100] Shrivastava N., Nag J.K., Pandey J., Tripathi R.P., Shah P., Siddiqi M.I. (2015). Homology modeling of NAD^+^-dependent DNA ligase of the Wolbachia endosymbiont of *Brugia*
*malayi* and its drug target potential using dispiro-cycloalkanones. Antimicrob. Agents Chemother..

[101] Chauleau M., Shuman S. (2016). Kinetic mechanism and fidelity of nick sealing by *Escherichia coli* NAD^+^-dependent DNA ligase (LigA). Nucleic Acids Res..

